# Eleven generations of selection for the duration of fertility in the intergeneric crossbreeding of ducks

**DOI:** 10.1186/1297-9686-41-32

**Published:** 2009-03-31

**Authors:** Yu-Shin Cheng, Roger Rouvier, Hsiao-Lung Liu, Shang-Chi Huang, Yu-Chia Huang, Chung-Wen Liao, Jui-Jane Liu Tai, Chein Tai, Jean-Paul Poivey

**Affiliations:** 1 Livestock Research Institute, Council of Agriculture, Hsin-Hua, Tainan, 71246 Taiwan; 2 INRA, UR631, Station d'Amélioration Génétique des Animaux, BP 52627, 31326 Castanet-Tolosan, France; 3 Southern Taiwan University of Technology, Tainan, 71005 Taiwan; 4 CIRAD, UR18, Systèmes d'élevage, 34398 Montpellier, France

## Abstract

A 12-generation selection experiment involving a selected line (S) and a control line (C) has been conducted since 1992 with the aim of increasing the number of fertile eggs laid by the Brown Tsaiya duck after a single artificial insemination (AI) with pooled Muscovy semen. On average, 28.9% of the females and 17.05% of the males were selected. The selection responses and the predicted responses showed similar trends. The average predicted genetic responses per generation in genetic standard deviation units were 0.40 for the number of fertile eggs, 0.45 for the maximum duration of fertility, and 0.32 for the number of hatched mule ducklings' traits. The fertility rates for days 2–8 after AI were 89.14% in the S line and 61.46% in the C line. Embryo viability was not impaired by this selection. The largest increase in fertility rate per day after a single AI was observed from d5 to d11. In G12, the fertility rate in the selected line was 91% at d2, 94% at d3, 92% at days 3 and 4 then decreased to 81% at d8, 75% at d9, 58% at d10 and 42% at d11. In contrast, the fertility rate in the control line showed an abrupt decrease from d4 (74%). The same tendencies were observed for the evolution of hatchability according to the egg set rates. It was concluded that selection for the number of fertile eggs after a single AI with pooled Muscovy semen could effectively increase the duration of the fertile period in ducks and that research should now be focused on ways to improve the viability of the hybrid mule duck embryo.

## Introduction

The mule duck is the major commercial source of duck meat (soup or roasted) and is produced by crossing Tsaiya, Pekin or Kaiya (crossbred Pekin × White Tsaiya) ducks with Muscovy drakes. The reproductive efficiency of ducks has been successfully improved over the last twenty years in Taiwan by using artificial insemination (AI) [1]. This is also a popular method in France where male mule ducks are force-fed to produce fatty liver and the females are used for meat production [2], and in Europe, Vietnam and southeast China for meat production [3-5]. Thus in the last few decades, it has become common practice in many countries worldwide, to use AI as a reproduction technique for mule duck production.

Unfortunately, owing to the short duration of fertility in such intergeneric crossbreeding, the ducks have to be inseminated twice a week in order to maintain the fertility rate [6-8]. It would be economically beneficial if the female could be inseminated once instead of twice a week and if the fertility rate could be increased. The aim of the selection experiment was therefore to increase the duration of fertility in order to reduce the frequency of AI required. Previous results in domestic fowl had demonstrated the feasibility of selecting for a longer fertile period [9,10]. Thereafter, Tai *et al*. [11] found that the best selection criterion for duration of fertility in the Brown Tsaiya female duck seemed to be the number of fertile eggs laid between the 2^nd ^and 15^th ^day after a single AI with pooled Muscovy semen. Therefore, in 1992, the Livestock Research Institute (LRI), Hsinhua, Tainan, Taiwan began a selection experiment to increase the number of fertile eggs (F) in the Brown Tsaiya female duck after a single AI with pooled Muscovy semen in one selected and one control (unselected) line [12]. Fertility was measured by candling the eggs on the 7^th ^day of incubation. The genetic parameters for the duration of fertility in Brown Tsaiya duck were estimated from the data obtained from the selected and control lines up to the 5^th ^generation of selection [13]. The selection responses for number of fertile eggs up to the 7^th ^generation of selection were analyzed [14]. The effects of selection on duration of fertility and its consequences on hatchability over 10 generations of selection were characterized using logistic curves to adjust fertility and hatchability rates as a function of number of days after AI [15]. Reports were published throughout the selection experiment [14,16,15].

It is the usual practice to inseminate with pooled semen for producing mule ducklings. However in generation 12 the objective for an experiment was to evaluate fertility of Muscovy drakes after single AI with individual semen.

As far as we know no full analyses of the direct and correlated effects of long-term selection experiments in ducks have been published. This study analyses the direct response to the selection on number of fertile eggs after a single AI of Brown Tsaiya duck with pooled Muscovy semen and correlated responses on the maximum duration of fertility, number of hatched mule ducklings, duration of fertility and hatchability for eleven generations of selection.

## Methods

### Animals and developing lines

One hundred and six Brown Tsaiya LRI no. 2 female ducks and 28 Brown Tsaiya LRI no. 2 drakes, originating from a Brown Tsaiya Line 105 studied for laying traits and developed at the Ilan branch of the Livestock Research Institute (LRI), were used as foundation stock (G0) [17,18]. Foundation birds were assumed to be unrelated and not inbred. In the first generation (G1), 165 ducks and 117 drakes were divided into two groups. The selected line (S) consisted of 48 ducks and 23 drakes bred from different parents, and with the highest predicted breeding values according to the BLUP animal model, for the number of fertile eggs at candling (F). The control line (C) consisted of 46 ducks and 20 drakes selected with near average predicted breeding values in each family. These two groups were used to produce the subsequent generation (G2). The first hatch in G1 was on February 16, 1992 and the last one in G12 was on January 4, 2005. Both lines were maintained simultaneously under standardized conditions at the LRI experimental farm in Hsinhua, Tainan. In total, 1438 males and 2602 females in the S line, 1097 males and 2105 females in the C line were measured and recorded respectively. Generations were kept separate and the generation interval was one year. In the S line, the percentage selected was between 40% and 20.2% in females and between 10.9% and 20.8% in males.

#### Selected line

In the S line, male and female ducks in each generation were selected by applying the BLUP animal model and operating a truncation selection on the highest values for number of fertile eggs from the 2^nd ^to 15^th ^day after AI (3 times). The following model was used to determine the breeding values of the selected trait, as described in Cheng [12]:

**y **= **Xb **+ **Z**_1 _**a **+ **Z**_2 _**p **+ **e**

where **y **= vector of observations;

**b **= vector of fixed effects of hatching date;

**a **= vector of random genetic effect with E(**a**) = 0, Var(**a**) = **A **, where **A **is the additive genetic relationship matrix of the animals,  = the additive genetic variance;

**p **= vector of random permanent environmental effect (3 times AI at 26, 29 and 32 weeks of age) with E(**p**) = 0, Var(**p**) = **I **, where **I **is the identity matrix,  = the variance of permanent environmental effects;

**e **= vector of random residual effects with E(**e**) = 0, Var(**e**) = **I **, where  = the variance of random residual effects;

**X**, **Z**_1 _and **Z**_2 _= design matrices relating the elements **b**, **a **and **p **to the observations.

For each generation, an additive genetic relationship matrix was established by taking into account all the ancestors of the selection candidates back to the foundation stock. Duck performance in all generations (from G1) was also taken into account.

The genetic parameter estimates used for G1 to G3 were h^2 ^= 0.34 [11] and repeatability r = 0.47 (estimated from G1 data). These values were h^2 ^= 0.29 and r = 0.40 [12] for G4 to G6, and h^2 ^= 0.26 and r = 0.36 [13] from G7 to G12. The breeding values of the candidates to be selected were computed, using a software written by Poivey [19] for G1 to G3, and with the PEST program [20] thereafter. The schedule was to select 20 males for G1 to G8, 12 males from G9 to G12 and 60 females in each generation so that one male could be mated with 3 or 5 females to produce the offspring to be measured in the following generation. From G2 to G12, it was scheduled to have 4 full-sister daughters of each selected dam. The number of full-brother sons of each selected dam was about 2 on average.

#### Control line

**Figure 1:**
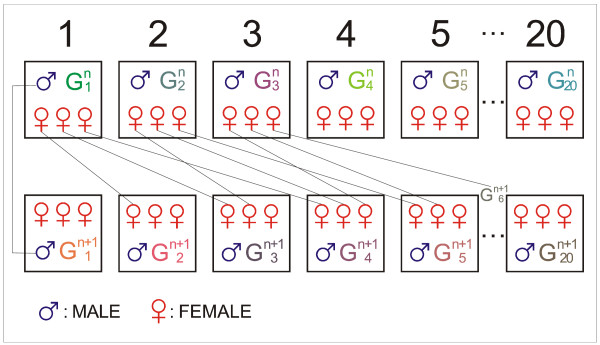
**Mating plan of control line**.

The plan was to maintain the control line by selecting 20 sires and 60 dams (3 dams per sire). One son of each sire was randomly chosen to replace his father and one daughter of each dam was randomly chosen to replace her mother, for mating according to the rotational scheme shown in Figure 1[21]. In the mating plan, constitutive groups of breeders in the control line for the generation G^n+1 ^were divided into 20 groups. The three females in the group  were from three different sire groups (m = 1 to 20). The 20 males stayed in their groups. One sire gave one male and one dam gave one female, the sire of group  was the son of group , his mother was one of three dams in group . The three dams in group  gave three females, the first went to the group , the second to the group  and the third to the group .

### Management and experiment

The ducklings were raised in floor pens and fed a diet containing 19% CP and 2925 Kcal ME kg^-1 ^from 0 to 4 wk followed by a diet containing 13% CP and 2830 Kcal ME kg^-1^, from 5 to 15 wk. They were transferred to individual cages when they had laid their first egg. During the laying period, the ducks were fed a diet containing 20% CP and 2810 Kcal ME kg^-1^. Drinking water and feed were provided *ad libitum *throughout the experimental period. At 26, 29, and 32 weeks of age, the ducks were artificially inseminated with 0.05 mL of pooled semen from 10 to 15 Muscovy drakes of line 302 from LRI, Ilan Station [22,23].

In addition, at G12, ducks were artificially inseminated at 36, 39 and 42 weeks of age with individual semen from 23 Muscovy drakes, adopting the ratio of one male for six females. Purpose of this experiment was to evaluate the genetic effects of Muscovy drakes on duration of fertility (unpublished results). So in G12, individual and pooled semen were used on the same ducks at different ages.

After a single AI, eggs were collected from day 2 to 15 for G1 to G6, and from day 2 to 18 for G7 to G12. They were stored for 7 days and 9 days respectively in the incubator to ensure egg set. Fertility was measured by candling the eggs after 7 days of incubation, and the number of live-hatched ducklings was recorded. Data regarding the number of eggs set (Ie), the number of fertile eggs at candling (F), the total number of dead embryos (M), the maximum duration of fertility from the 2^nd ^day after AI up to the day of the last fertile egg (Dm, in number of days), and the number of hatched mule ducklings (H) were collected. A new generation of ducks was produced by pedigree mating, and pedigree hatching was carried out in each generation thereafter.

### Statistical analysis

The elementary statistical parameters (means and variances) of the phenotypic values were obtained using the SAS^® ^procedure [24]. Any unintentional selection was detected by calculating the selection differentials on the breeding values of F in the C line in each generation, from the differences between the averages of birds randomly chosen as parents and from all birds measured in that generation. The inbreeding coefficients were calculated in each generation for the females and males of each line, by using a SAS^® ^procedure [24]. The direct cumulated selection responses and correlated selection responses were measured as the differences between the phenotypic performance averages of the ducks in the S and C lines. Their variances were calculated by taking into account the variances of the error measurements and of the genetic drift [25-28].

The predicted genetic responses to selection on F were estimated from the within generation line difference (S-C) for the average predicted breeding values for each of the five traits in female ducks. These breeding values were calculated in a 5-trait analysis using the BLUP methodology applied to an individual animal model and previously described for a single trait. These multiple-trait BLUP animal model values were calculated by grouping the records of all five traits together for the selected and control lines from G1 to G12, using the PEST 3.1 package [20,29], with a performance file containing 11721 records and a pedigree file of 7096 ducks. The genetic and phenotypic parameters for the five traits used to estimate these breeding values, were taken from Poivey *et al*. [13]. For simplification, the estimated parameters were used to calculate the approximate standard errors for the generation S-C differences for each trait, given that the breeding values were computed in univariate analyses [30].

When the fertility or hatchability rates per egg set of the S and C lines in the same generation were plotted as a function of the number of days after AI, the resulting curves were adjusted to logistic functions, in which parameter τ was the time in days of half maximal fertility or hatchability [15,31].

## Results

### Percentage of selection

Over the 11 generations of selection, the average percentage of selected females was 28.9% and of selected males was 17.05%. The unintentional selection differential, which occurred over the 11 generations of selection in the C line was small (-1.09 fertile eggs). It should be noted that the ducks of the S and C lines came from the same hatches in all generations, except G2. In G1 some parents were used to constitute both the S and C lines. In G2, the S line birds were born on 10/02/1993 and 09/03/1993, whereas the C line birds were born on 07/04/1993. Although some AI were performed during the same period in both groups, others were not and this could have led to some inaccuracy in the measurement of selection response in G2.

### Inbreeding coefficients

Table 1 shows the mean inbreeding coefficients for males and females of the S and C lines, for each generation. Individuals of the foundation stock were assumed to be unrelated and not inbred. Therefore, the average inbreeding coefficient in G1 was 0. This was also the case in G2, due to the rotational nature of the mating plan in the C line. In contrast, full-sib and half-sib matings were avoided in the S line. More than one male from a given family with the highest predicted breeding values according to the BLUP animal model for the number of fertile eggs at candling (F) could be used to produce the subsequent generation as selected line. Thereafter, the inbreeding coefficient increased more quickly in the S line than in the C line, as could be expected, but remained moderate: the means in G12 were 0.154 and 0.068 for the males and 0.156 and 0.074 for the females in the S and C line respectively.

**Table 1:** Mean of inbreeding coefficients in males and females of S and C lines

Generation	S line		C line	
	Male	Female	Male	Female

G1	0	0	0	0
G2	0	0	0	0
G3	0.018	0.017	0.0078	0.0067
G4	0.036	0.041	0.025	0.022
G5	0.047	0.053	0.034	0.034
G6	0.065	0.067	0.038	0.040
G7	0.084	0.082	0.048	0.047
G8	0.106	0.106	0.063	0.060
G9	0.108	0.112	0.066	0.065
G10	0.117	0.118	0.071	0.065
G11	0.140	0.142	0.059	0.059
G12	0.154	0.156	0.068	0.074

### Selection responses and predicted genetic responses

The genetic parameters (heritabilities and genetic correlations) of the five traits Ie, F, M, Dm, and H were used to calculate the multiple-trait BLUP animal model values for each trait for all measured females from generations G1 to G12. These genetic parameters had been estimated in the conceptual base population [13].

**Figure 2:**
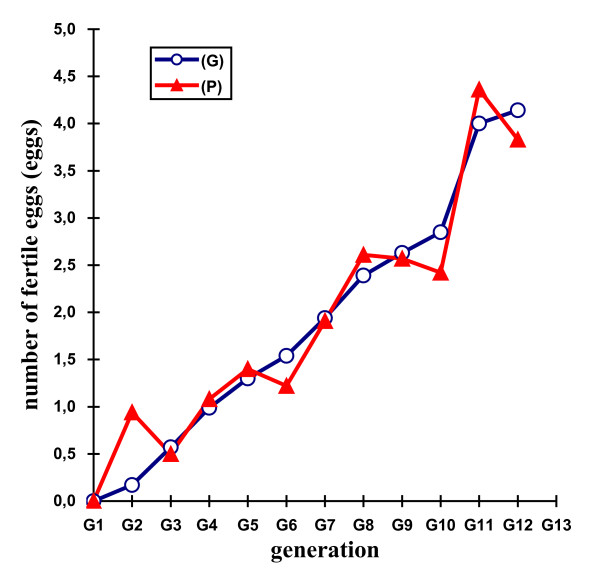
**Differences in number of fertile eggs at candling**.

**Figure 3:**
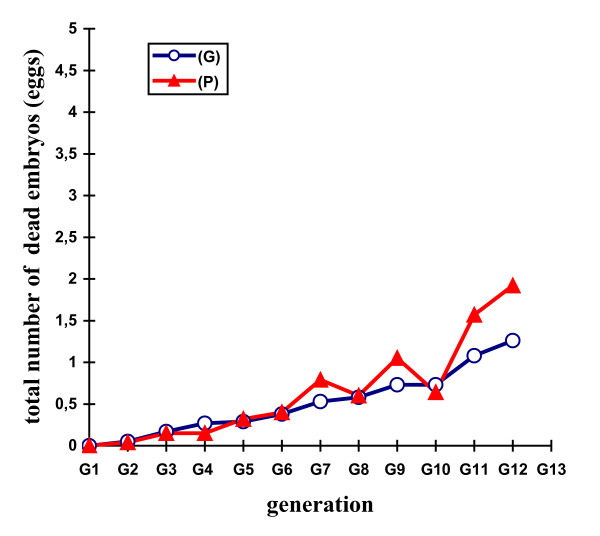
**Differences in total number of dead embryos**.

**Figure 4:**
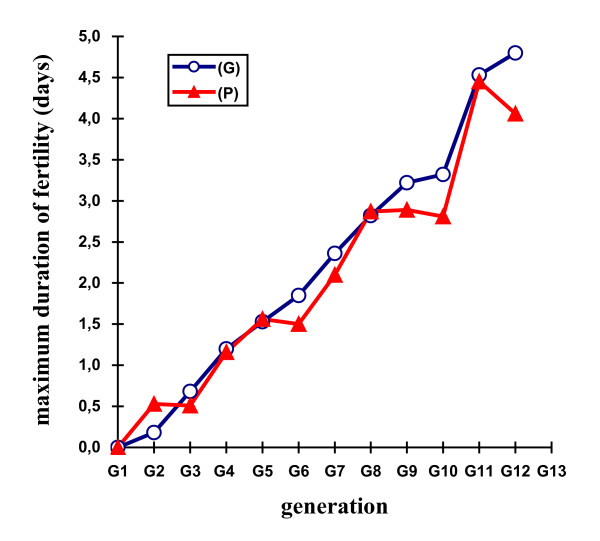
**Differences in maximum duration of fertility**.

**Figure 5:**
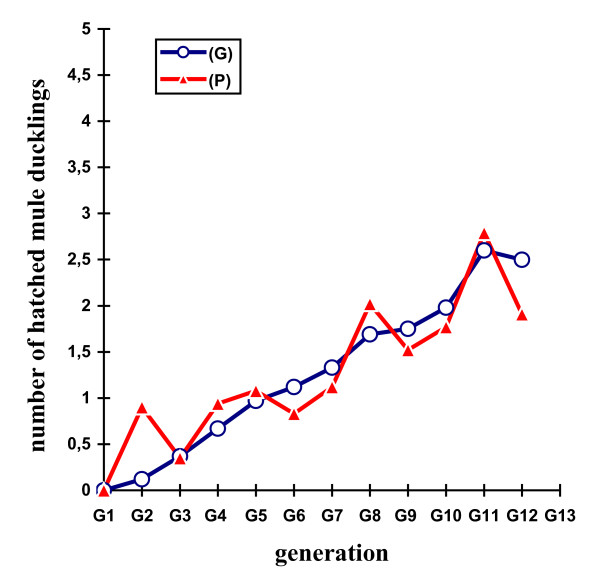
**Differences in number of hatched mule ducklings**.

**Figure 6:**
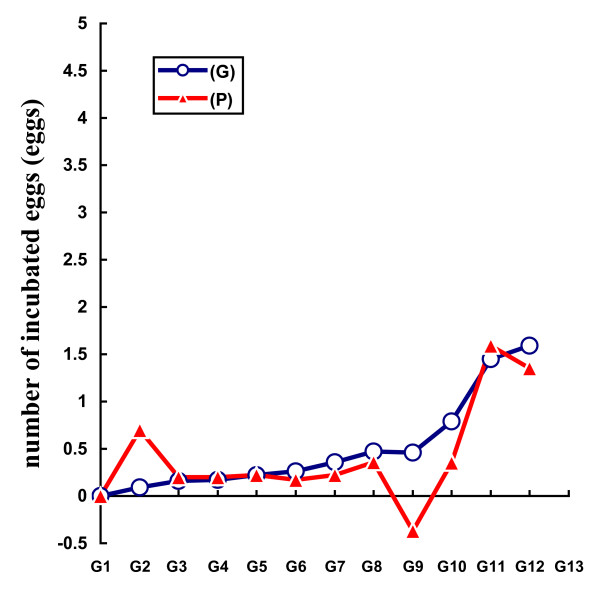
**Differences in number of incubated eggs between selected (S) and control (C) lines for the phenotypic (P) and predicted genetic (G) values across 11 generations of selection**.

Table 2 shows the mean selection responses (with standard errors) and predicted genetic responses (with standard errors) for the F, Ie, M, Dm, and H traits across the 11 generations of selection. Figures 2, 3, 4, 5 and 6 show the trends in the selection responses and predicted genetic responses of F, M, Dm, H and Ie. The two responses were similar, except that the former showed greater fluctuation between generations. The selection responses were highly significant for the selected trait and for the correlated traits Dm and H at G4. The correlated selection response for M and Ie became significant at G5 and G11 respectively. At G11, the mean selection response and the mean predicted genetic response were very close, being 4.36 and 4.00 respectively for F, 1.57 and 1.08 for M, 4.45 and 4.53 for Dm, 2.79 and 2.60 for H. These genetic increases at G11 were represented as a percentage of the average traits in G1: 103% for F, 85% for M, 79% for Dm, and 116% for H. Table 3 shows the mean (and standard deviation) of the phenotypic values and selection response S-C (P) of Brown Tsaiya females for the F, Ie, M, Dm, and H traits after artificial insemination with individual semen from the 23 Muscovy drakes for G12 in the selected line (S) and control line (C) at 36–42 weeks of age. The mean selection response S-C (P) was 3.46 for F, 0.85 for M, 3.68 for Dm and 2.59 for H.

**Table 2:** Mean of the traits in G1, selection responses (SR) mean ± standard errors, mean of predicted genetic responses (PGR) ± standard errors for the five traits

Generation		G1	G2	G3	G4	G5	G6	G7	G8	G9	G10	G11	G12
Trait		Mean											
F	SR	4.23	0.94 ± 0.21	0.50 ± 0.27	1.08 ± 0.32	1.40 ± 0.36	1.22 ± 0.41	1.91 ± 0.43	2.61 ± 0.50	2.57 ± 0.67	2.42 ± 0.70	4.36 ± 0.64	3.83 ± 0.84
	PGR		0.17 ± 0.058	0.57 ± 0.060	0.99 ± 0.065	1.30 ± 0.061	1.54 ± 0.055	1.94 ± 0.041	2.39 ± 0.060	2.63 ± 0.064	2.85 ± 0.057	4.00 ± 0.055	4.14 ± 0.094

Ie	SR	11.83	0.70 ± 0.16	0.20 ± 0.20	0.20 ± 0.26	0.22 ± 0.28	0.17 ± 0.31	0.22 ± 0.33	0.36 ± 0.47	-0.37 ± 0.53	0.35 ± 0.90	1.59 ± 0.50	1.35 ± 0.68
	PGR		0.09 ± 0.026	0.16 ± 0.028	0.17 ± 0.041	0.22 ± 0.032	0.26 ± 0.032	0.36 ± 0.026	0.47 ± 0.037	0.46 ± 0.038	0.79 ± 0.044	1.45 ± 0.034	1.59 ± 0.047

M	SR	1.84	0.04 ± 0.08	0.15 ± 0.09	0.15 ± 0.11	0.32 ± 0.11	0.40 ± 0.14	0.79 ± 0.13	0.60 ± 0.15	1.05 ± 0.29	0.64 ± 0.26	1.57 ± 0.32	1.92 ± 0.40
	PGR		0.05 ± 0.019	0.17 ± 0.017	0.27 ± 0.019	0.29 ± 0.013	0.38 ± 0.016	0.53 ± 0.014	0.58 ± 0.019	0.73 ± 0.019	0.73 ± 0.022	1.08 ± 0.018	1.26 ± 0.022

Dm	SR	5.63	0.53 ± 0.22	0.51 ± 0.28	1.16 ± 0.34	1.56 ± 0.38	1.50 ± 0.43	2.10 ± 0.45	2.87 ± 0.50	2.89 ± 0.66	2.81 ± 0.74	4.45 ± 0.63	4.06 ± 0.79
	PGR		0.18 ± 0.069	0.68 ± 0.072	1.20 ± 0.071	1.53 ± 0.063	1.85 ± 0.059	2.36 ± 0.045	2.82 ± 0.067	3.22 ± 0.069	3.32 ± 0.062	4.53 ± 0.060	4.80 ± 0.095

H	SR	2.39	0.90 ± 0.17	0.35 ± 0.21	0.94 ± 0.25	1.08 ± 0.28	0.83 ± 0.31	1.12 ± 0.33	2.02 ± 0.36	1.52 ± 0.57	1.77 ± 0.59	2.79 ± 0.55	1.91 ± 0.73
	PGR		0.12 ± 0.043	0.37 ± 0.048	0.67 ± 0.052	0.97 ± 0.051	1.12 ± 0.044	1.33 ± 0.032	1.69 ± 0.047	1.75 ± 0.048	1.98 ± 0.042	2.60 ± 0.038	2.50 ± 0.070

F = number of fertile eggs at candling (7^th ^day of incubation); Ie = number of eggs set; M = total number of dead embryos; Dm = maximum duration of fertility (d); H = number of hatched mule ducklings

**Table 3:** Means ± standard deviations of phenotypic values and selection response S-C (P) of Brown Tsaiya females following AI with the individual semen of the Muscovy drake for G12 in the S and C lines at 36–42 weeks of age

	S line	C line	S-C
Ducks	n = 150	n = 83	
F	7.59 ± 2.58	4.13 ± 1.96	3.46 ± 0.84
Ie	14.10 ± 1.86	12.95 ± 2.73	1.15 ± 0.49
M	2.15 ± 1.75	1.30 ± 1.37	0.85 ± 0.36
Dm	9.04 ± 2.56	5.36 ± 2.40	3.68 ± 0.79
H	5.43 ± 2.41	2.84 ± 1.80	2.59 ± 0.67

F = number of fertile eggs at candling (7^th ^day of incubation); Ie = number of eggs set; M = total number of dead embryos; Dm = maximum duration of fertility (d); H = number of hatched mule ducklings

Table 4 shows the mean (and standard deviation) of fertility and hatchability rates for days 2–15 or days 2–8 after a single AI for the S and C lines in G12 at 26–32 weeks of age (pooled semen) and 36–42 weeks of age (individual semen). The F/Ie, H/Ie percentages in the S and C lines were significantly different for days 2–15 and 2–8 after AI. The hatchability rate calculated as the ratio H/F was slightly higher in the C line than in the S line but this difference was not statistically significant. Due to the effect of an abnormal operation of the incubator, a significant age effect on H/F % was apparent in the S line, especially at G12 *i.e*. it was larger at 36–42 weeks of age than at 26–32 weeks of age (73.0% versus 60.62%). A larger H/F % value at 36–42 weeks of age than at 26–32 weeks of age was also apparent in the C line (73.06% versus 69.26%), but the difference was not significant.

**Table 4:** Mean ± standard deviation of fertility and hatchability rates for days 2–15 or days 2–8 after a single AI for S and C lines in G12 at 26–32 weeks of age (pooled semen) and 36–42 weeks of age (individual semen)

	Days 2–15 after AI			Days 2–8 after AI		
Line	Fertility rate	Hatchability rates		Fertility rate	Hatchability rates	

	F/Ie%	H/Ie%	H/F%	F/Ie%	H/Ie%	H/F%

S	59.98^a ^± 4.00	36.24^a ^± 3.92	60.43^a ^± 3.99	89.14^a ^± 2.50	54.03^a ^± 4.07	60.62^a ^± 3.99

C (26–32 W)	33.80b ± 5.19	23.60^b ^± 4.66	69.80^a ^± 5.04	61.46^b ^± 5.34	42.57^b ^± 5.43	69.26^a ^± 5.06

S	58.15^a ^± 4.02	42.31^a ^± 4.03	72.60^a ^± 3.64	88.88^a ^± 2.57	64.92^a ^± 3.90	73.00^a ^± 3.62

C (36–42 W)	32.72b ± 5.15	23.91^b ^± 4.68	73.07^a ^± 4.87	62.26^b ^± 5.32	45.49^b ^± 5.47	73.06^a ^± 4.87

Ie = number of eggs set; F = number of fertile eggs at candling (7^th ^day of incubation); H = number of hatched mule ducklings; two different subscripts (a, b) in a column indicate significant differences (P < 0.05)

### The parameter τ of the logistic curves

**Figure 7:**
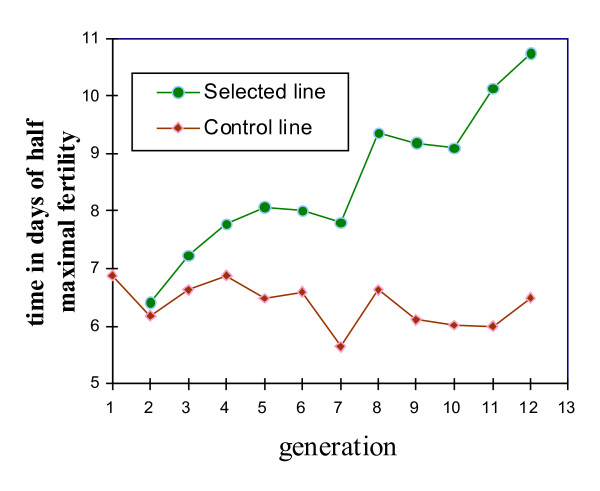
**Evolution of τ, time in days of half maximal fertility, across the generations of selection, in the selected (S) and control (C) Brown Tsaiya duck lines**.

**Figure 8:**
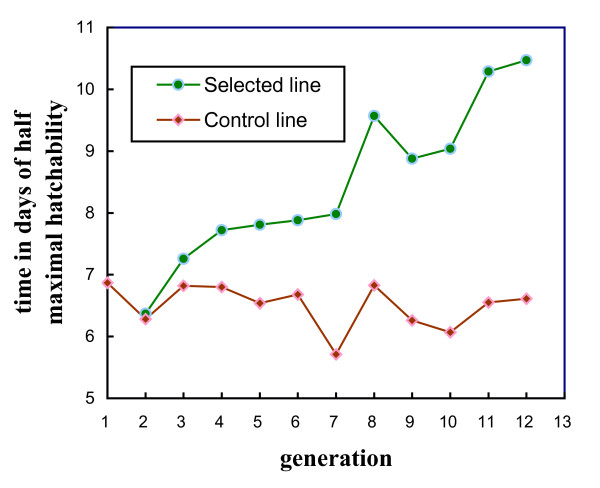
**Evolution of τ, time in days of half maximal hatchability according to eggs set, across the generations of selection, in the selected (S) and control (C) Brown Tsaiya duck lines**.

**Figure 9:**
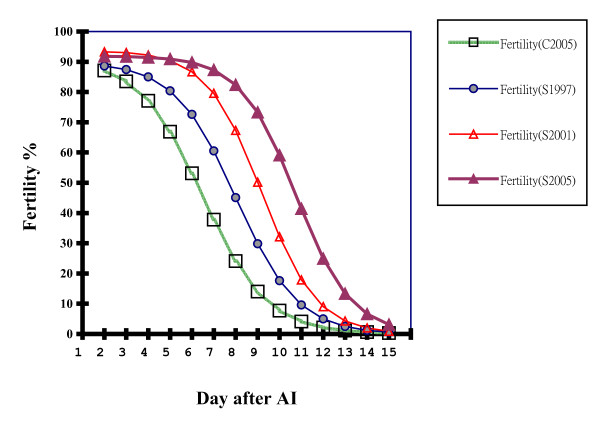
**Duration of fertility after a single artificial insemination (AI) with pooled Muscovy semen of selected (1997, 2001 and 2005) and control (2005) Brown Tsaiya lines**. Solid lines for 2005 represent the functions of logistic curves. y(x) = 91.90 (1+e^-0.7874(10.745-x)^)^-1 ^for the selected line(S2005) and y(x) = 91.25 (1+e^-0.6797(6.489-x)^)^-1 ^for the control line(C2005).

Figure 7 shows the evolution of τ, time in days of half maximal fertility, of selected (S) and control (C) Brown Tsaiya duck lines across the generations of selection, and the S-C differences. The S-C differences were significant from G3 onwards. They were as high as 4.26 days in G12 (10.75 d. and 6.49 d. for the S and C lines, respectively) showing that the selection response was positive. Figure 8 shows the evolution of τ, the time of half maximal hatchability. The S-C differences were also significant from G3 onwards. They increased up to 3.86 days (10.47 d. and 6.61 d. for the S and C lines, respectively) showing that the correlated selection response was also positive. Figure 9 shows the adjusted logistic curves and the durations of fertility according to the egg set rates in 1997 (G6), 2001 (G9) and 2005 (G12) for the S line and in 2005 for the C line. The R^2 ^were >0.99 indicating the goodness of fit. In the S line (in G12) the fertility rates were 91% at d2, above 90% up to d5 and higher than 80% from d6 to d8. From d9 onwards they began to decrease (75%), to (58%) on d10 and 3% on d15 (Table 5). In contrast, the fertility rates in the C line, which were 85% at d2, showed an abrupt decrease from d4 (74%) onwards: *i.e*. d5 (69%), d6 (52%), d7 (36%), d8 (26%), d10 (8%) and 0.5% at d15. A similar pattern was observed for hatchability rates (Table 5). Consequently, the logistic curve still had the same form but was moved to the right by selection.

**Table 5:** Fertility rates (%) and hatchability rates (%) in selected (S) and control (C) Brown Tsaiya duck lines of G12, as a function of the number of days following a single artificial insemination (AI) with pooled Muscovy semen, and values of Student-Fisher t (1)

	**Duck line**	**Nb of ducks**	**Number of days after AI**													
			**2**	**3**	**4**	**5**	**6**	**7**	**8**	**9**	**10**	**11**	**12**	**13**	**14**	**15**
F/Ie	S	150	91	94	92	92	86	87	81	75	58	42	26	12	4	3
	C	83	85	87	74	69	52	36	26	13	8	5	1.6	1.5	0	0.5
	t(1)		1.3	1.7	3.4	4.2	5.5	8.6	9.5	12.1	10.0	7.9	6.4	3.5	2.5	1.6
H/Ie	S	150	53	60	59	61	56	48	41	44	35	27	16	7	2	1.2
	C	83	59	58	54	48	38	26	15	9	7	5	1	2	0	0
	t(1)		-0.9	0.3	0.7	1.9	2.7	3.5	4.6	6.8	5.8	5.1	4.7	1.9	1.7	1.3

Fertility rate (%): F/Ie *100, ratio of number of fertile eggs (F) to the number of eggs set (Ie); Ie per day varied between 395 and 417 eggs in the S line, between 179 and 209 eggs in the C line

Hatchability rate (%): H/Ie *100, ratio of number of hatched mule ducklings (H) to the number of eggs set (Ie)

t(1): difference of fertility and hatchability rates between S and C duck lines, divided by standard deviation of the difference

## Discussion

In avian species the fertile period has been defined as the interval after sperm deposition during which a female can lay fertile eggs. The length of the fertile period is dependent on sperm storage in the tubules at the utero-vaginal junction where the spermatozoa are released for upward transport towards the infundibulum for ova fertilization [32]. The purpose of this selection experiment was to investigate what genetic progress could be made to extend the duration of fertility in the Brown Tsaiya duck. The selection was carried out using an animal model and the BLUP of breeding values. The selection experiment was continued up to 12 generations using the same methodology of selection already discussed in Cheng *et al*. [14]. In addition the durations of fertility and hatchability were determined and their correlated responses to selection on F were analyzed. The selection responses were calculated, using the common method of calculating selection responses by taking the differences between the average phenotypic values for the S and C lines across the generations of selection [25,33]. Sorensen and Kennedy [30] described an alternative way of estimating response to selection based on the mixed model approach, as the phenotypic trend can be further divided into genetic and environmental trends. We therefore estimated the genetic trends by averaging the multiple-trait BLUP animal model values for each trait in each generation and determined the differences between the S and C lines.

The measured selection responses and the calculated predicted genetic responses were found to be similar. This indicated the adequacy of the data representation model with no confounding with environmental trends and the accuracy of the genetic parameter estimates in the base population. Given the large variability in selection response, especially of H, we have chosen to discuss the predicted genetic response. The genetic progress in F measured by the predicted genetic response was significant *i.e*. 4.40 genetic standard deviations in total or 40% of the genetic standard deviation per generation. The correlated genetic progress in Dm and H was also significant, *i.e*. 4.89 and 3.56 genetic standard deviations in total, or 45% and 32% of the average genetic standard deviation per generation, respectively. The frequency of embryo mortality was not increased by selection. These results are consistent with the estimated genetic parameters, thereby showing a high genetic correlation between F and Dm (0.92), H (0.91) and between Dm and H (0.82). In contrast to results obtained in the chicken hen [34,35] and according to the genetic parameter estimates, our results showed that selection on F seemed to be more effective in increasing H than direct selection of that trait. Brun *et al*. [36] reported heritabilities of 0.25 and 0.23 for F, 0.17 and 0.13 for H, and 0.27 and 0.16 for Dm in pure breeding INRA44 duck line and intergeneric crossbreeding, respectively. Our result can be explained by the fact that the heritability of F is greater than that of H (0.26 versus 0.19) and the genetic correlation between F and H is 0.91.

This study showed that the selection of F through 11 generations had major correlative effects on parameter τ of the logistic curves, which fitted the daily variations (d2-d15) in fertility rates (F/Ie) and hatchability rates (H/Ie). The S-C differences represented selection responses to the duration of fertility and hatchability which were correlated with the selection response of F. Selection for F modified the evolution of the fertility and hatchability rates, as a function of time after a single AI of the Tsaiya duck with pooled Muscovy semen mainly by increasing the time of half maximal fertility and hatchability rates. The largest increases in the fertility rates per day after single AI were between d5 and d11. Selection for F also had correlated effects on the maximum fertility rates, but these were smaller than the effect on fertility duration. Moreover, the fertility rate in the selected line was over 90% from d2 to d5 and above 80% until d8. The same tendencies were observed for changes in the evolution of hatchability rates, showing that embryo viability was not impaired. Consequently, in accordance with Brillard *et al*. [37] it is suggested that selection on F acted by increasing the storage capacity of spermatozoa, which remained able to fertilize the ova for longer. In addition, the increased duration of fertility when selecting on F was not deleterious to embryo viability. The overall fertility (F/Ie) and hatchability (H/Ie) rates at days 2–8 after AI were higher in the S line than in the C line. The embryonic viability rates in the C line (73.1%) and S line (73.0%), measured from the hatchability of fertile eggs (H/F), were not statistically different for G12 (36–42 weeks of age), confirming the results for G8 and G11 [38,14,15]. The differences in hatchability of fertile eggs (H/F) between the S and C lines over the 11 generations of selection were not statistically different either.

On the basis of the results of Tai *et al*. [11], a long-term selection experiment on F, with a selected and a control line, was begun in 1992. Analysis of this experiment after 11 generations of selection revealed a selection response for F (3.83 eggs), with correlated selection responses for increasing H (1.91 ducklings) and maximum duration of the fertile period (4 days), with no increase in embryo mortality rate. The genetic progress in F measured by the selection response was 2.77 genetic standard deviations or 39.6% of the genetic standard deviation per generation in G8 and 4.07 genetic standard deviations or 37% of the genetic standard deviation per generation in G12. The correlated selection response in Dm was also increased from 2.93 to 4.14 genetic standard deviations between G8 and G12. There was no increase in H in G12 compared to G8, due to an electric cut off problem in the incubator and M was increased. However there was a large variability of selection response in H. In G11 the selection response in H (2.79) was higher than in G8 (2.02). In G12 the correlated selection response on H measured at 36, 39 and 42 weeks of age (2.59) was a more relevant value.

The realized selection response for F can be compared with the theoretically expected one if selection has been done with the conventional combined selection index although that prediction of response is valid in principle for only one generation of selection. The expected selection response on F, according to the accuracy of the combined selection index on F, would be higher than the realized one. That can be explained by variation of response due to random genetic drift and sampling errors [13]. In addition there was a loss in selection intensity especially because some animals with a high-predicted breeding value were discarded from reproduction to avoid full sib and half sib mating.

Selection to extend the fertile period was shown to be feasible [14,15]. The present results confirm the absence of a selection plateau in responses up to the 11^th ^generation. Selection was effective in increasing the number of ova that could be fertilized after a single AI with pooled Muscovy semen, and consequently the number of eggs able to develop a viable embryo. These changes considerably increased the maximum duration of the fertile period, and the physiological effects now need to be investigated. Selection brought about a correlated increase in fertility and hatchability rates according to egg set, especially for days 2–8 after AI, thereby demonstrating the feasibility of selection for a single AI per week in this strain of laying duck. This did not produce a concomitant increase in the rate of embryonic death, (previously thought to occur in fowl) which would have impaired the benefits of selection. Thus fertilization of the ova would seem to be a key point in the intergeneric crossbreeding of ducks [39,40]. Nevertheless, the total mortality rate in relation to the number of fertile eggs was high (23 to 36% (G11)). It would therefore be useful to continue this selection experiment and study the long-term effects on fertility and embryo viability. A better understanding of the consequences of selection was obtained by comparing the fertility rate curves [31] according to the number of days after AI in the S and C lines. The genetic variability of viability in ducks needs to be determined to evaluate the possibilities of improving mule embryo viability. The results obtained here might depend on the use of Brown Tsaiya, which is a laying duck. Nonetheless, it should be feasible to select for an extension of the fertile period in meat-type ducks such as the Peking breed, which is being used effectively as parents for commercial mule ducks. Furthermore, research can now be focused on ways to improve the viability of the hybrid mule duck embryo.
